# Antithrombotic Therapy in Patients with Peripheral Artery Disease: A Focused Review on Oral Anticoagulation

**DOI:** 10.3390/ijms22137113

**Published:** 2021-07-01

**Authors:** José Miguel Rivera-Caravaca, Anny Camelo-Castillo, Inmaculada Ramírez-Macías, Pablo Gil-Pérez, Cecilia López-García, María Asunción Esteve-Pastor, Esteban Orenes-Piñero, Antonio Tello-Montoliu, Francisco Marín

**Affiliations:** 1 Department of Cardiology, Hospital Clínico Universitario Virgen de la Arrixaca, University of Murcia, Instituto Murciano de Investigación Biosanitaria (IMIB-Arrixaca), CIBERCV, 30120 Murcia, Spain; anjo134@gmail.com (A.C.-C.); minmaculadarm@gmail.com (I.R.-M.); pablogilperez1@gmail.com (P.G.-P.); cecilopezgarcia23@gmail.com (C.L.-G.); masunep@gmail.com (M.A.E.-P.); atellomont@hotmail.com (A.T.-M.); fcomarino@hotmail.com (F.M.); 2 Liverpool Centre for Cardiovascular Science, University of Liverpool and Liverpool Heart & Chest Hospital, Liverpool L7 8TX, UK; 3 Department of Biochemistry and Molecular Biology-A, University of Murcia, Instituto Murciano de Investigación Biosanitaria (IMIB-Arrixaca), CIBERCV, 30120 Murcia, Spain; eorenes@um.es

**Keywords:** peripheral artery disease, anticoagulation therapy, rivaroxaban, antiplatelet therapy

## Abstract

Peripheral artery disease (PAD) is a major cause of morbidity and mortality but it is usually underdiagnosed and undertreated. Patients with PAD present dysregulated procoagulant, anticoagulant, and fibrinolytic pathways leading to arterial and venous thrombosis. The risk of several ischemic-related complications could be mitigated with appropriate antithrombotic therapy, which plays a central role in all types of PAD. For years, antiplatelets have been indicated in patients with symptomatic PAD or those who have undergone revascularization. Unfortunately, a non-negligible proportion of patients with PAD will suffer from adverse events during the follow-up, even despite proper medical therapies for the prevention of PAD complications. Thus, there is room for improving clinical outcomes in these patients. Given the implication of both, primary and secondary hemostasis in arterial thrombosis and the pathophysiology of PAD, the combination of antiplatelets and anticoagulants has emerged as a potential antithrombotic alternative to antiplatelets alone. In this narrative review article, we have highlighted the most recent evidence about antithrombotic therapy in PAD patients, with a special focus on oral anticoagulation. Certainly, COMPASS and VOYAGER PAD trials have shown promising results. Thus, rivaroxaban in combination with aspirin seem to reduce cardiovascular outcomes with a similar bleeding risk compared to aspirin alone. Nevertheless, results from real-world studies are needed to confirm these observations, and other trials will provide novel evidence about the safety and efficacy of emerging anticoagulant agents.

## 1. Introduction

Peripheral artery disease (PAD) is a major cause of morbidity and mortality. It is well known that coagulation activation and endothelial stimulation are significantly increased in patients with PAD, and these factors are intimately related with the severity of the arterial disease [1]. Besides, elevated platelet activation, altered fibrinogen levels, thrombin generation, and fibrin turnover are typical in PAD [2]. These patients, especially those with critical limb threatening ischemia, present decreases in natural anticoagulants (proteins C and S) and coagulation factors FIX, FXI, and FXII [3]. Thus, dysregulated procoagulant, anticoagulant, and fibrinolytic pathways lead to arterial and venous thrombosis in   patients with PAD [4]. The contribution of atherosclerosis, and ultimately of thrombosis, to ischemic risk in PAD has formed the basis for treatment with antithrombotic therapy [5].

### Relevance of Peripheral Artery Disease

PAD comprises a diverse group of disorders that lead to progressive stenosis, occlusion, or aneurysmal dilation of the aorta and its non-coronary branch arteries, including the carotid, upper extremity, visceral, and lower extremity arterial branches [6,7]. The prevalence of PAD is approximately 12% in the adult population, affecting >200 million people worldwide, and this prevalence is age-dependent, being males affected slightly more than females [8,9,10].

The most frequent presentation of PAD is the lower extremity artery disease (LEAD). Patients with LEAD may suffer from cramping, tiredness, numbness, or weakness in the legs, hips, thighs, or calf muscles during certain activities. Indeed, ~50% of LEAD patients present symptoms such as intermittent claudication or other symptoms that may evolve to acute limb ischemia [11]. However, this disease is often underdiagnosed because it also may be present without ischemic symptoms related to the legs or even with atypical symptoms.

The risk of PAD increases with established cardiovascular risk factors, such as hypertension, dyslipidaemia, smoking habit, and diabetes [12,13,14,15]. Indeed, other cardiac diseases including coronary artery disease (CAD), heart failure, and atrial fibrillation (AF) are frequent in patients with PAD, especially in patients with LEAD. For example, the coexistence of AF, CAD, and PAD is usual and related with a higher risk of worse clinical outcomes [16,17,18,19]. Thus, the coexistence of PAD with other cardiac conditions needs a multidisciplinary approach with important therapeutic and prognostic implications.

Patients with LEAD are at high risk for major adverse cardiovascular events (MACE) over the long term, and also at high risk for major adverse limb events (MALE) such as severe limb ischaemia,   gangrene, intermittent claudication, functional impairment,   and amputation with   mortality of ~50% at 1-year [20]. In addition, one of the worst outcomes of PAD is the decrease in the overall quality of life and well-being due to atypical leg pain and claudication. These symptoms lead to sedentary lifestyle because the discomfort and pain limit movements, walk, and exercise practice. Hospitalization rates are also increased in patients with PAD [21]. Nevertheless, the major negative consequence is a markedly increased cardiovascular morbidity (mainly by myocardial infarction and stroke) and mortality (cardiovascular and all-cause) [22]. Importantly, in the Framingham Study, 75% of PAD patients died from cardiovascular events, observing that in patients with intermittent claudication, mortality was 2–3 times higher [23].

With these key clinical outcomes in mind, evidence-based management of PAD   includes cholesterol reduction, blood pressure and glucose level control, physical exercise therapy, and smoking cessation. Appropriate pharmacological therapy is also encouraged, by using both antiplatelets and oral anticoagulants [24]. Indeed, the risk of many of the cited complications could be mitigated with the right antithrombotic therapy, playing a central role in all types of PAD. In the present review, we focus on antithrombotic therapy in LEAD, as this is the most common condition.

## 2. Pathophysiology of Thrombosis and Hemostasis in Peripheral Artery Disease

Atherosclerosis triggers stenosis and vasoconstriction, thus producing ischemic symptoms in PAD. It disrupts homeostasis and favors pro-thrombotic responses in the vasculature, platelet activation, and aggregation. For this reason, it could be said that atherosclerosis is the root cause of the pathophysiology process in PAD [25]. 

**Figure 1:**
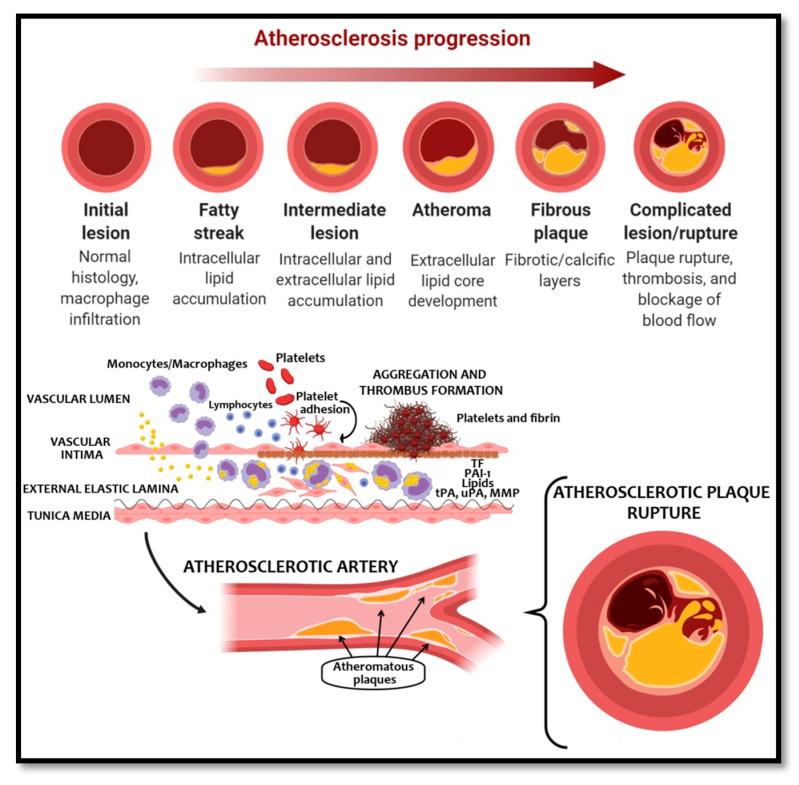
Atherosclerosis progression, thrombus formation, and atherosclerotic plaque rupture.

In turn, this pro-thrombotic state produces an acceleration of atherosclerosis that even in the absence of acute occlusive phenomenon, produces ischemic symptoms due to progressive stenosis of the vessel. Ultimately, atherosclerosis may lead to thrombosis by arterial occlusion if an atherosclerotic plaque is ruptured, which aggravates the process, increases vascular involvement, and leads to adverse clinical outcomes with more limited prognosis (Figure 1).

The main initiator of clot formation is the tissue factor (TF), which is released by endothelial cells after a vascular damage (for example, in the case of a ruptured or ulcerated atherosclerotic plaque) [26]. Indeed, elevated levels of TF have been shown in patients with PAD [27]. TF gains access to proconvertin (factor VII in plasma), and together form the TF/FVIIa complex. The TF/FVIIa complex activates small amounts of FIX and FX. On TF-exposing cells, FXa associates with FVa to form the prothrombinase complex. In the prothrombinase complex, prothrombin is converted into thrombin (factor IIa), which then transforms fibrinogen into fibrin [28,29,30].

On the other hand, the strongest natural inhibitor of the extrinsic coagulation system is the tissue factor pathway inhibitor (TFPI), which in combination with concentrations of TF/FVIIa complex, regulates the duration of this phase. FIXa is not inhibited by TFPI but is slowly inhibited by antithrombin. Antithrombin inactivates thrombin, binding it to a stable inactive thrombin–antithrombin complex (TAT). Increased TAT complex concentrations are a marker of intensified thrombin generation, and therefore a biomarker for thrombosis. FIXa does not act on the TF-bearing cell but migrates and binds to the surface of activated platelets which are localized at the injury site [31,32]. The small quantity of thrombin is the key factor for further thrombin and fibrin production. 

In parallel, platelets respond to traumatic vascular injury by adhesion, activation, and aggregation. Indeed, in atherosclerosis diseases such as PAD, platelets suffer changes in morphology, function, and activation degree. They do not only adhere and aggregate at the site of the vascular lesion but also contribute to assemble of the prothrombinase complex, thus promoting thrombin generation and fibrin clot formation [25]. Importantly, fibrin clot itself exhibits significant thrombin-binding potential since the concentration of free thrombin in blood plasma decreases due to its binding not only to fibrinopeptide A/B cleavage sites on the fibrinogen molecule but also by binding to fibrinogen through an anion-binding site (exosite 1). In addition, antithrombin I also shows a significant affinity to the D nodules of fibrin (ogen) molecules containing the γ chain variant termed γ′ [33].

Simultaneously, thrombin activates platelets inducing the release of FV and FVa from their α-granules. A positive feedback loop is initiated, whereby thrombin activates circulating FV and releases FVIII from von Willebrand factor (vWF), and activates it. FVa and FVIIIa bind to platelet surfaces and serve as cofactors for large-scale thrombin generation. Thrombin also activates FXI and FXI binds to the surfaces of activated platelets [31]. In fact, thrombin is the most potent platelet activator [34], which explains why the selective inhibition of the cellular actions of thrombin through antagonism of PAR-1, the main thrombin receptor on platelets, has been explored [35]. In the propagation phase, the FVIIIa/FIXa and the FVa/FXa complexes (prothrombinase) are formed on the surface of activated platelets and accelerate the generation of FXa and thrombin, respectively. In addition, FXIa bound to the platelet surface, activates FIX to form an additional FVIIIa/FIXa complex. This complex activates Fx (FXa) which rapidly binds with FVa on the platelet surface. Prothrombinase complex initiates a burst of thrombin, which converts fibrinogen to fibrin. Soluble fibrin monomers polymerize to form fibrin protofibrils, which are stabilized by FXIIIa to form a solid fibrin network that in turn stabilizes [36]. In this point, it should be noted that altered fibrinogen levels are associated with thrombosis. Thus, raised fibrinogen levels increase rates of ischemic events [37,38], but there are also cases of decreased fibrinogen levels (hypofibrinogenemia) in the context of a specific mutation which might be associated with thrombosis [39]. Indeed, in quantitative fibrinogen disorders, some free thrombin remains in the circulation with its level directly depending on the fibrinogen plasma level. Low plasma levels of fibrinogen in hypofibrinogenemic patients can partially suppress thrombin activity and this is considered to be the largest prothrombotic trigger in patients with quantitative fibrinogen disorders [40].

In addition, the prothrombotic character of platelets is influenced by the presence of numerous plasma hemostasis factors in their membrane microvesicles (platelet microparticles), which contributes to the activation of the coagulation system [26]. Glycoprotein (GP) Ib-IX-V interacts with vWF on exposed subendothelial components, and tethered platelets bind to collagen through GPVI and integrin αIIβ1. Following the initial tethering of platelets to the vessel wall, subsequent adhesion results in signal transduction within platelets and their flattening. Platelet agonists released by circulating platelets recruit and activate further platelets [41,42]. During this process, penetration of monocytes/macrophages and lymphocytes into damaged vessels increases and contributes to the destabilization of the atherosclerotic plaque [43,44]. 

On the other hand, fibrinolysis is a process that occurs as a compensatory mechanism to coagulation and its main function is limiting hypercoagulability. The reaction is catalyzed by tissue plasminogen activator (tPA) or urokinase plasminogen activator (uPA) present in circulating blood, which ultimately leads to the formation of plasmin from plasminogen. This enzyme, with important proteolytic properties, dissolves the fibrin clot into fibrin degradation products (such as D-dimer). The activation of fibrinolysis is inhibited by plasminogen activator inhibitor type 1 (PAI-1) released from endothelium and platelets [26,29]. If the balance of fibrinolytic activators to their inhibitors is disturbed, this may result from the depletion of PAI-1 [45] and in patients with PAD, previous studies have demonstrated and increased fibrinolysis and fibrinolysis inhibition [46,47,48]. 

Finally, it should be noted that inflammation is a key mediator of the atherosclerotic process and likely contributes to the limb manifestations of PAD. Thus,   inflammation markers such as C-reactive protein, interleukin-6   or   thromboxane are also increased in PAD patients [47,49,50].

The implication of both, primary and secondary hemostasis in arterial thrombosis, in turn activating both circulating platelets and the coagulation cascade, has formed the basis of the combined blockade of platelets and coagulation in stable atherosclerosis by using antiplatelets and anticoagulants.

## 3. Antithrombotic Therapy in PAD

Classically, the central element of antithrombotic management in patients with PAD has been the use of antiplatelets. These drugs prevent limb-related and cardiovascular events in LEAD patients, and therefore long-term antiplatelet therapy is recommended by current European Society of Cardiology and American Heart Association/American College of Cardiology (AHA/ACC) guidelines in patients with symptomatic LEAD [6,7]. In the absence of concomitant atherosclerotic disease or other indication for oral anticoagulation (OAC), both guidelines strongly recommend the use of single antiplatelet therapy. However, it remains unclear which specific antiplatelet agent should be used, although   clopidogrel is an effective alternative to   acetylsalicylic acid (ASA)   for prevention of cardiovascular events in symptomatic   LEAD. Actually, the ESC guidelines suggest its use over ASA in these patients (Class of recommendation IIb; level of evidence B) [6]. In contrast, LEAD patients with documented CAD or revascularization may benefit from a more intense antithrombotic therapy. Thus, dual antiplatelet therapy (DAPT) with ASA and a P2Y_12_ inhibitor may be considered for a short period of time in LEAD patients with recent acute coronary syndrome (ACS) and/or percutaneous coronary intervention (<1 year), or after lower extremity revascularization [6,7].

Despite these recommendations, an important proportion   of patients with newly diagnosed PAD are not treated conforming to guidelines, and at least 33% of these patients do not receive any antithrombotic therapy [51]. In addition, although medical prevention of PAD complications has increased over time, a non-negligible proportion of patients will still suffer adverse events during the follow-up, particularly those related to lower extremities such as major amputations [52].

  For this reason, there is room for improving clinical outcomes in PAD, and other antithrombotic alternatives have been tested. Thus, the role of OAC is still controversial   even though older trials investigated the efficacy and safety of OAC in PAD. For example, the Warfarin Antiplatelet Vascular Evaluation (WAVE) trial assigned patients with PAD to combination therapy with an antiplatelet agent and a vitamin K antagonist (VKA) (warfarin or acenocoumarol with target INR 2.0–3.0) or to antiplatelet therapy alone. The results of this trial showed that the combination of a VKA and antiplatelet therapy was not more effective than antiplatelet therapy alone in preventing major cardiovascular complications and was associated with an increase in life-threatening bleeding in this patient population [53].   Thus, current guidelines recommend OAC   therapy only if there is a concomitant indication and may be combined with SPAT in the context of a recent revascularization [6]. However, the emergence of direct-acting oral anticoagulants (DOACs) in the field of LEAD has shown promising results; and these drugs have potential for changing the current paradigm in the management of PAD.

### 3.1. Where Are We Now? Using Rivaroxaban in Peripheral Artery Disease

The most investigated DOAC for the reduction of cardiovascular outcomes in patients with previous atherosclerosis is rivaroxaban, given its pleiotropic actions in cardiovascular prevention. FXa has a dual-pathway function by decreasing thrombin generation and potentially showing complementary favorable activity, such as anti-inflammatory effects and endothelial protection [54,55]. To date, rivaroxaban is the only DOAC that has successfully undergone phase III evaluation in patients with ACS. Thus, in the ATLAS ACS 2-TIMI 51 trial, rivaroxaban reduced the risk of the composite endpoint of cardiovascular death, myocardial infarction, or stroke in patients with a recent ACS but also increased the risk of major bleeding and intracranial hemorrhage [56]. However, the GEMINI-ACS-1 trial showed that the combination of low-dose rivaroxaban (2.5 mg twice daily, commonly known as “vascular dose”) with a P2Y_12_ inhibitor for the treatment of ACS patients had similar risk of clinically significant bleeding as ASA plus a P2Y_12_ inhibitor [57]. 

These promising results have been taken to the field of PAD (Table 1). The ‘Rivaroxaban for the Prevention of Major Cardiovascular Events in Coronary or Peripheral Artery Disease (COMPASS)’ trial included more than 27,000 patients with stable CAD or PAD at high risk for ischemic events. Participants were randomized to rivaroxaban (2.5 mg twice daily) plus ASA (once daily), rivaroxaban (5 mg twice daily) alone, or ASA (once daily) alone. After a mean follow-up of 23 months, the rate of the composite of cardiovascular death, stroke, or myocardial infarction, was lower with low-dose rivaroxaban plus ASA than with ASA alone (HR 0.76; 95% CI 0.66–0.86; *p* < 0.001), as were total mortality, coronary heart disease mortality, and cardiovascular mortality. Although major bleeding was increased in the combination arm (HR 1.70; 95% CI, 1.40–2.05; *p* < 0.001), fatal or critical organ bleeding was not [58].

In a sub-analysis of patients with stable peripheral or carotid artery disease, the combination of rivaroxaban and ASA reduced major adverse cardiovascular (MACE) (HR 0.72; 95% CI 0.57–0.90; *p* = 0.005) and limb events (MALE) (HR 0.54; 95% CI 0.35–0.82; *p* = 0.004) when compared to ASA alone among the 7470 patients with PAD included in the COMPASS population. In a similar way to the overall trial results, there was an increase in the risk of major bleeding in the rivaroxaban plus ASA arm (HR 1.61; 95% CI 1.12–2.31; *p* = 0.009) but not an increase in the risk of fatal or critical organ bleeding [59]. Focusing specifically in patients with PAD included in the COMPASS, MALE events were significantly associated with higher risk of subsequent hospitalizations, amputations, and death. Of note, the combination of rivaroxaban 2.5 mg twice daily and ASA reduced the incidence of MALE by 43% (*p* = 0.01), total vascular amputations by 58% (*p* = 0.01), peripheral vascular interventions by 24% (*p* = 0.03), and all peripheral vascular outcomes by 24% (*p* = 0.02), compared with ASA alone [60]. In addition, a recent sub-analysis of the COMPASS trial in patients with symptomatic LEAD, among patients with either high-risk limb presentations or high-risk comorbidities, rivaroxaban plus ASA compared with ASA alone was associated with an estimated 4.2% (95% CI 1.9%–6.2%) absolute risk reduction for MACE or MALE, including major amputation, at 30 months, resulting in a net clinical benefit of 3.2% (95% CI 0.6%–5.3%) [61]. These results derived from the COMPASS trial highlighted the potential role of rivaroxaban for secondary prevention in patients with PAD. 

More recently, the ‘Vascular Outcomes Study of ASA Along With Rivaroxaban in Endovascular or Surgical Limb Revascularization for Peripheral Artery Disease (VOYAGER PAD)’ trial aimed to evaluate the efficacy of rivaroxaban added to antiplatelet therapy to reduce major cardiovascular and limb ischemic vascular outcomes in patients with PAD undergoing a lower-extremity revascularization [62]. A total of 6564 patients were randomized to receive rivaroxaban (2.5 mg twice daily) plus ASA or placebo plus ASA. At 3-years of follow-up, there was a significant reduction of the composite of acute limb ischemia, major amputation for vascular causes, myocardial infarction, ischemic stroke, or death from cardiovascular causes, in patients in the rivaroxaban arm (HR 0.85; 95% CI 0.76–0.96; *p* = 0.009). In terms of safety, major bleeding was significantly higher in the rivaroxaban group according to the ISTH major bleeding definition (HR 1.42; 95% CI, 1.10–1.84; *p* = 0.007), but not according to the TIMI major bleeding definition (HR 1.43; 95% CI, 0.97–2.10; *p* = 0.070) [63].

Despite this evidence from trials, there is always the remaining question of whether results will be generalizable to ‘real-world’ patients. Although further investigation is needed from the real-world setting, an analysis of the “REduction of Atherothrombosis for Continued Health (REACH)” Registry demonstrated that COMPASS-eligible patients represent a substantial fraction of PAD patients (68.4%) usually managed in routine clinical practice, which suggest that this trial has good external applicability [64]. However, other studies will   assess ischemic and bleeding outcomes in patients with CAD, PAD, or both, receiving rivaroxaban 2.5 mg twice daily plus ASA. This is the case of the ‘Xarelto plus Acetylsalicylic acid: Treatment patterns and Outcomes in patients with Atherosclerosis (XATOA)’ registry, which will include up to 10,000 patients from at least 400 centers in 22 countries [65].  

### 3.2. Role of Other DOACs

  As mentioned above,   rivaroxaban attracted most of the interest for patients with CAD and/or PAD. However, there are other DOACs that have been recently tested in patients with atherosclerotic diseases. For example, the “Edoxaban in Peripheral Arterial Disease (ePAD)” investigated the safety and potential efficacy of edoxaban and ASA versus clopidogrel and ASA for 3 months following endovascular treatment. Among the 203 patients included, there was a similar risk for major and life-threatening bleeding events in the two groups, with a lower non-significant incidence of restenosis/re-occlusion in the edoxaban arm compared to the clopidogrel arm at 6 months (30.9% vs. 34.7%; RR 0.89, 95% CI 0.59–1.34, *p* = 0.643) [66].

The ‘Management of Myocardial Injury After Noncardiac Surgery Trial (MANAGE)’ trial assessed the impact of dabigatran to prevent major vascular complications among patients with myocardial injury after non-cardiac surgery. The trial randomized 1754 patients to receive dabigatran (*n* = 877) or placebo (*n* = 877) and demonstrated that the risk of the primary efficacy outcome (the composite of   vascular mortality and non-fatal myocardial infarction, non-haemorrhagic stroke, peripheral arterial thrombosis, amputation, and symptomatic venous thromboembolism)   was significantly lower in patients assigned dabigatran than placebo (HR 0.72, 95% CI 0.55–0.93; *p* = 0.0115) with a non-significant differences in the risk of the primary safety outcome (composite of life-threatening, major, and critical organ bleeding) (HR 0.92, 95% CI 0.55–1.53; *p* = 0.76) [67]. 

Finally, the ‘Efficacy and Safety of Apixaban in Reducing Restenosis and Limb Loss in PAD Patients (AGRIPPA)’ is an   exploratory trial that will evaluate the efficacy and safety of apixaban 2.5 mg twice daily plus ASA compared with DAPT (clopidogrel plus aspirin) in 200 patients with critical limb ischemia undergoing endovascular infrapopliteal revascularization followed for 12 months. The aim of this trial is to prove the concept of an alternative antithrombotic regimen for these patients which allow testing the hypothesis in a future large randomized clinical trial [68].  

## 4. The Future Is Coming: FXI and FXII as Targets for Novel Anticoagulants

Despite DOACs having been demonstrated to be a good option for thromboprophylaxis in different settings, the search for anticoagulation alternatives with even better safety profile, has moved forwards focusing on other components of the contact system [69]. Thus, FXII and FXI have emerged as new targets for potentially safer anticoagulants. Both factors have shown a role in thrombosis, with the advantage that FXII deficiency is not associated with bleeding diathesis, and patients with FXI deficiency rarely experience spontaneous bleeding [70,71,72].

Studies to date in this field have shown interesting results. For example, a preclinical study assessed the antithrombotic activity of Ixodesricinus contact phase inhibitor (Ir-CPI), a protein which specifically inhibits both factors XIIa and XIa, in animal contact phase-initiated thrombosis models, including cardiopulmonary bypass. During cardiopulmonary bypass, Ir-CPI was as efficient as unfractionated heparin in preventing clot formation within the extracorporeal circuit and maintained physiological parameters during and post-surgery. Contrary to heparin, Ir-CPI did not promote bleeding [73].

  However, new evidence is expected from randomized clinical trials. Hence, the BMS-986177, and oral FXIa inhibitor, is being compared with placebo in the AXIOMATIC-SSP trial (NCT03766581), a phase II trial for secondary prevention in patients with stroke or high-risk TIA. In this study, all patients receive BMS-986177 + ASA+ clopidogrel (or placebo) for 21 days followed by aspirin alone thereafter. The primary outcome is the composite of new ischemic stroke during the treatment period and new covert brain infarction detected by brain imaging at 90 days. The study is estimated to be completed in 2022 [74].

Specifically in atherosclerotic disease, the   PACIFIC-AMI (NCT04304534) trial will compare the efficacy and safety of BAY2433334 once daily, another orally available FXIa inhibitor, with placebo on top of dual antiplatelet therapy (ASA + clopidogrel) for prevention of MACE in 1600 patients with acute MI. The primary efficacy endpoint is any of composite of cardiovascular death, MI, stroke, or stent thrombosis, whereas the primary safety endpoint is the occurrence of ‘Bleeding Academic Research Consortium (BARC)’ bleeding definition type 2, 3, and 5. The trial is currently ongoing and results are expected also for 2022 [75].

## 5. Final Remarks

**Figure 2:**
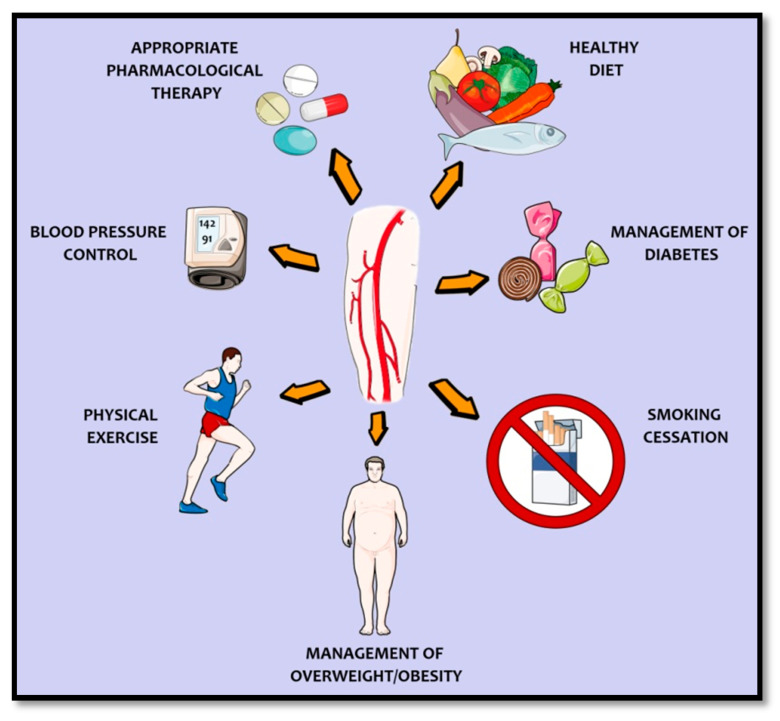
Integrated and multidisciplinary management of peripheral artery disease.

Patients with PAD comprise a high cardiovascular risk population. However, many of these patients are still underdiagnosed and undertreated. Part of the integrated and multidisciplinary management of PAD includes smoking cessation, healthy diet, weight loss, and regular physical exercise, as well as antihypertensive, lipid-lowering, and antidiabetic agents, as is summarized in Figure 2. Within this pharmacological approach, antithrombotic drugs have gained special attention.

For years, antiplatelets have been indicated if PAD patients are symptomatic or have undergone revascularization, whereas OAC therapy was recommended only in the presence of a concomitant indication. However, the publication of recent trials in this field has opened new possibilities. Rivaroxaban is now a real option for PAD patients, and data from these trials show that its combination as a vascular dose with ASA may reduce cardiovascular outcomes with a similar bleeding risk compared to ASA alone. Although more evidence from real-world studies is needed, observations to date have the potential of changing our current clinical practice.

In the upcoming years, further studies will investigate the safety and efficacy of emerging anticoagulant agents, and this could help to find novel molecules with the ability to reduce thrombotic complications with a relative reduced bleeding risk. These include, for example, drugs   which target clotting factors XI and XII, or their activated forms—XIa and XIIa—or even   other Factor XIa inhibitors.

## Figures and Tables

**Table 1:** Summary of results from randomized clinical trials using rivaroxaban in the context of peripheral artery disease.

Title	Sample Size	Arms	Main Efficacy Outcomes	Main Safety Outcomes
COMPASS	27,395	Rivaroxaban (2.5 mg twice daily) plus aspirin (100 mg once daily) (N = 9152)	Primary outcome: Cardiovascular death, stroke, or myocardial infarction • Rivaroxaban plus aspirin vs. Aspirin alone: HR 0.76 (95% CI 0.66–0.86), *p* < 0.001 • Rivaroxaban vs. Aspirin: HR 0.90 (95% CI 0.79–1.03), *p* = 0.12	Major bleeding • Rivaroxaban plus aspirin vs. Aspirin alone: HR 1.70 (95% CI 1.40–2.05), *p* < 0.001 • Rivaroxaban vs. Aspirin: HR 1.51 (95% CI 1.25–1.84), *p* < 0.001
		Rivaroxaban (5 mg twice daily) (N = 9117)		Fatal bleeding or symptomatic bleeding into critical organ • Rivaroxaban plus aspirin vs. Aspirin alone: HR 1.34 (95% CI 0.95–1.88), *p* = 0.09 • Rivaroxaban vs. Aspirin: HR 1.58 (95% CI 1.13–2.19), *p* = 0.006
		Aspirin (100 mg once daily). (N = 9126)		
COMPASS (LEAD patients only)	6391	Rivaroxaban (2.5 mg twice daily) plus aspirin (100 mg once daily) (N = 2139)	Major Adverse Limb Events: • Rivaroxaban plus aspirin vs. Aspirin alone: HR 0.57 (95% CI 0.37–0.88), *p* = 0.01 • Rivaroxaban vs. Aspirin: HR 0.71 (95% CI 0.47–1.06), *p* = 0.07	Major Bleeding • Rivaroxaban plus aspirin vs. Aspirin alone: HR 1.61 (95% CI 1.09–2.36), *p* = 0.01 • Rivaroxaban vs. Aspirin: HR 1.60 (95% CI 1.09–2.36), *p* = 0.02
		Rivaroxaban (5 mg twice daily) (N = 2129)		Fatal bleeding or symptomatic bleeding into a critical organ or surgical site bleeding requiring re-operation. • Rivaroxaban plus aspirin vs. Aspirin alone: HR 1.32 (95% CI 0.71–2.42), *p* = 0.38 • Rivaroxaban vs. Aspirin: HR 1.30 (95% CI 0.70–2.40), *p* = 0.41
		Aspirin (100 mg once daily). (N = 2123)		
VOYAGER PAD	6564	Rivaroxaban (2.5 mg twice daily) plus aspirin (100 mg once daily) (N = 3286)	Primary outcome: acute limb ischemia, major amputation for vascular causes, myocardial infarction, ischemic stroke, or cardiovascular death • Rivaroxaban plus aspirin vs. Placebo plus aspirin: HR 0.85 (95% CI 0.76–0.96), *p* = 0.009	Major bleeding (TIMI definition) • Rivaroxaban plus aspirin vs. Placebo plus aspirin: HR 1.43 (95% CI 0.97–2.10), *p* = 0.07
		Placebo plus aspirin (100 mg once daily) (N = 3278)		Intracranial or fatal bleeding • Rivaroxaban plus aspirin vs. Placebo plus aspirin: HR 0.91 (95% CI 0.47–1.76)

LEAD = lower extremity artery disease.

## Data Availability

Not applicable.
